# Are evidence-based vasectomy surgical techniques performed in low-resource countries?

**DOI:** 10.12688/gatesopenres.12986.2

**Published:** 2019-07-22

**Authors:** Michel Labrecque

**Affiliations:** 1 CHU de Québec-Université Laval Research Centre, Population Health and Optimal Health Practices, 1050 Chemin Sainte-Foy, local K0-03, Quebec City, Quebec, G1S 4L8, Canada; 2 Department of Family and Emergency Medicine, Laval University, Quebec City, Quebec, Canada

**Keywords:** Male Sterilization, Surgical Procedure, Vasectomy, Family Planning Services, Developing Countries, Practice Guideline, Quality of Health Care, Guideline Adherence

## Abstract

**Background:  **Research evidence published 10 to 15 years ago has shown that the type of vasectomy surgical technique performed can influence the effectiveness and the safety of the procedure.  The objective of this study was to determine if evidence-based vasectomy surgical techniques are integrated in the vasectomy programs of selected low-resource countries.

**Methods: **The surgical techniques recommended to perform the two steps of the vasectomy procedure (isolation/exposition and occlusion of the vas deferens) were extracted from current evidence-based clinical practice guidelines. Documents describing male sterilisation standards and practice from Kenya, Rwanda, India, Nepal, Mexico, Honduras, Colombia and Haiti were reviewed to assess adequacy with international guideline recommendations.

**Results: **Best recommended techniques are 1) a minimally invasive technique including the no-scalpel technique (known as the no-scalpel vasectomy (NSV)) to isolate and expose the vas deferens, and 2) cautery of the mucosa of the vas preferably combined with interposition of the fascia (FI) to occlude the vas deferens. The NSV is largely adopted and performed to isolate the vas in selected low-resources countries. Ligation and excision (LE) of a small segment of the vas deferens combined with FI is the most common vas occlusion technique mentioned in the country standards. Cautery as recommended in the guidelines is seldom used in selected countries.

**Conclusions: **  Effective and adapted vasectomy vas occlusion techniques are available, but are still underused in many low-resource countries. Providing the most effective vasectomy surgical techniques increases users’ confidence and satisfaction regarding male sterilization and may lead to higher acceptability and uptake.

## Introduction

Vasectomy is generally regarded as a simple, safe, very effective, and highly cost-effective contraceptive method. In the early 2000s, randomized trials ^1, 2^, comparative studies ^3– 5^, systematic reviews ^6, 7^ and expert consultations ^8^ showed that specific surgical techniques are associated with better safety and effectiveness of the procedure. More recently published North American and European practice guidelines on vasectomy based their recommendations on these findings ^9– 12^.

Although the uptake of vasectomy is low in most low-resource countries, some have active vasectomy programs ^13^. The objectives of this study were to determine 1) which vasectomy surgical techniques are recommended in evidence-based practice guidelines to reduce surgical complications (bleeding and infections) and to maximize occlusion and contraceptive effectiveness, and 2) if these techniques are integrated in the vasectomy norms and standards, and current practice of targeted low-resource countries.

## Methodology

### Recommended techniques

The recommended techniques of the two surgical steps of the vasectomy procedure (isolation/exposition and occlusion of the vas deferens) were extracted by the author from the following vasectomy practice guidelines: the European Association of Urology (2012) ^9^, American Urological Association (2012, 2015) ^10^, the Faculty of Sexual & Reproductive Healthcare (FSRH), the standard-setting organisation for family planning and sexual health in United Kingdom (2104) ^11^, and the Canadian Urological Association (2016) ^12^. The level of evidence, strength of recommendation and the most relevant underlying evidence from systematic reviews supporting the recommendations was also extracted.

### Data from low-resource countries

A convenience sample of eight low-resource countries from Africa, Asia and America known by the author to provide vasectomy services on different scales was selected. India, Nepal, Mexico, and Colombia (through Profamilia, a non-profit non-governmental organisation) have large and structured vasectomy programs with thousands of men vasectomized each year while private or governmental smaller scale initiatives exist in Kenya, Rwanda, Honduras and Haiti.

For each country, the most recent document describing vasectomy techniques that should be used (national standards/norms) and/or that are performed was first identified through personal contact with individuals from or acquainted with vasectomy in selected countries. In addition, in order to validate the currency of documents retrieved, a Google search was performed twice, in spring 2018 and April 2019, using the name of the country, “vasectomy” or “male sterilization”, and key words from the title of documents already identified. No date limits were imposed. The retrieved Google search pages were scanned until no more related documents were found. PubMed or Google Scholar search was not performed because, as expected, none of the relevant documents initially retrieved was published as peer-reviewed article.

The surgical techniques recommended and/or commonly performed to isolate/expose (classic technique with a scalpel, NSV) and to occlude the vas (simple LE, LE+FI, cautery) in the selected countries were extracted from the retrieved documents. Additional information on the surgical techniques commonly performed as obtained by personal contact with key informants was also reported. Guideline recommendations were compared to and contextualized with vasectomy techniques performed in the selected countries.

## Results

### Guideline recommendations

Excerpts of recommendations from the four practice guidelines are presented in Table 1. Although the assessment of the evidence and the strength of the recommendations vary across the four guidelines, they all agree that a minimally invasive (MIV) technique including the no-scalpel technique (known as the no-scalpel vasectomy (NSV)) should be performed to isolate and expose the vas deferens. The criteria of a MIV technique are: 1) a skin opening of ≤10 mm, 2) minimal dissection of the vas and perivasal tissues, and 3) no use of skin sutures ^10^. Among the MIV techniques, NSV is the most studied. Two systematic reviews concluded that NSV - based on high-quality evidence - is significantly associated with a lower risk of surgical complications, namely bleeding and/or hematomas ^6, 7^.

**Table 1.** Recommendations for exposing and occluding the vas deferens from practice guidelines on vasectomy.

Guideline	Excerpts of recommendations	LE	SR
	*Vas isolation*		
EAU ^9^	The no-scalpel vasectomy technique of isolation of the vas deferens is associated with fewer early complications, such as infections, haematomas, and less postoperative pain.	-	-
AUA ^10^	Isolation of the vas should be performed using a minimally-invasive vasectomy (MIV) technique such as the no-scalpel vasectomy (NSV) technique or other MIV technique.	B *	S *
FSRH ^11^	A minimally invasive approach should be used to expose and isolate the vas deferens during vasectomy, as this approach results in fewer early complications in comparison to other methods.	A ^†^	R ^†^
CUA ^12^	NSV is associated with a significantly lower risk of postoperative complications (hematoma, pain, infection) than conventional vasectomy.	A-B ^‡^	R ^‡^
	*Vas occlusion*		
EAU ^9^	Early recanalisation can be decreased by cautery (with either thermal or electrocautery devices) of the vas deferens and by fascial interposition.	1a ^§^	A ^§^
AUA ^10^	The ends of the vas should be occluded by one of three divisional methods: Mucosal cautery (MC) with fascial interposition (FI) and without ligatures or clips applied on the vas; MC without FI and without ligatures or clips applied on the vas; Open ended vasectomy leaving the testicular end of the vas unoccluded, using MC on the abdominal end and FI; or by the non-divisional method of extended electrocautery.	C *	R *
FSRH ^11^	Cauterisation followed by division of the vas deferens, with or without excision, is associated with the lowest likelihood of early recanalisation (failure) when compared to other occlusion techniques. Division of the vas on its own is not an acceptable technique because of the associated failure rate. It should be accompanied by diathermy or ligation and fascial interposition.	A ^†^	R ^†^
CUA ^12^	Fascial interposition during vasectomy is associated with a significantly higher rate of azoospermia at three months than no interposition. Cautery of the vas is associated with a lower risk of failure (defined as >100 000/ml sperm in the ejaculate) than fascial interposition.	B ^‡^	R ^‡^

*AUA nomenclature: Grade A - high quality evidence: well-conducted randomized clinical trials (RCTs); exceptionally strong observational studies; Grade B - moderate quality evidence: RCTs with some weaknesses; generally strong observational studies; Grade C - low quality evidence: observational studies that provide conflicting information or design problems (such as very small sample size); Standards are directive statements that an action should (benefits outweigh risks/burdens) or should not (risks/burdens outweigh benefits) be undertaken based on Grade A or Grade B evidence. Recommendations are directive statements that an action should (benefits outweigh risks/burdens) or should not (risks/burdens outweigh benefits) be undertaken based on Grade C evidence. ^†^FSRH nomenclature: Grade A - Evidence based on randomised controlled trials; no strength of recommendations specified. ^‡^CUA nomenclature: Grade A - Based on clinical studies of good quality and consistency with at least one randomized trial; Grade B - Based on well-designed studies (prospective, cohort), but without good randomized clinical trials; Grade C - Based on poorer quality studies (retrospective, case series, expert opinion). ^§^EAU nomenclature: Grade 1a - Evidence obtained from meta-analysis of randomised trials; Recommendation A - Based on clinical studies of good quality and consistency addressing the specific recommendations and including at least one randomised trial. EAU, European Association of Urology; AUA, American Urological Association; FSRH, Faculty of Sexual & Reproductive Healthcare; CUA, Canadian Urological Association: LE, Level of evidence; SR, Strength of recommendation.

The guidelines also all agree that cautery of the mucosa of the vas lumen, preferably combined with interposing the fascia between the divided ends of the vas (fascial interposition (FI)), should be used to occlude the vas. Moderate-quality evidence from cohort studies showed that the “classical” ligation and excision (LE) technique consisting in putting two ligatures on the vas deferens and excising a small (1 cm) vas segment in between is associated with a high risk of occlusion failure based on post-vasectomy semen analysis, from 8 to 13% ^2, 3, 14– 16^, and contraceptive failure, from 4% after 3 years to 9% after 10 years ^17– 19^. Although a high-quality randomized trial ^2^ demonstrated that LE combined with FI on the testicular end can reduce the risk of failure by 50%, occlusion failure rate remained high at 5.9% (95% confidence interval 3.8% to 8.6%). Moderate quality evidence based on comparative cohort studies showed that combining cautery of the mucosa of the vas with either electro- or thermal-cautery, preferably combined with FI, is associated with the lowest risk of occlusion failure (<1%) ^10, 11^.

### National standards and practices

National standards and practices in targeted low-resource countries are described in Table 2 All countries selected have national standards/norms ^20– 27^; editions range from 2009 to 2018 ( Table 2).

The NSV is the preferred recommended technique to expose the vas in all eight countries. Only three countries, Kenya ^20^, India ^22^, and Haiti ^27^, mention that the “classic” technique, requiring a larger opening of the scrotal skin with a scalpel, is still acceptable.

**Table 2.** National standards and practices for exposing and occluding the vas deferens in selected low-resource countries. Countries with large vasectomy programs are in italics.

Country	Vas isolation		Vas occlusion		
	Classic	NSV	LE	LE+FI	Cautery
Kenya 2009 ^20^	S	S	S		P *
Rwanda 2015 ^21^		S		S	S
*India 2013* ^22^	S	S	S	S	
*Nepal 2010* ^23^		S	S	S	
*Mexico 2009* ^24^		S		S	
Honduras 2010 ^25^		S		S	
*Colombia 2018* ^26^		S		S	S
Haiti 2009 ^27^	S	S			P *

*personal communication with Dr. Doug Stein. NSV, no-scalpel vasectomy; LE, ligation and excision; FI, fascial interposition; S, country standards; P, Common practice but no written standards.

The most commonly vas occlusion technique recommended in the national standards is the LE combined with FI. Documents from India ^22^ and Nepal ^23^ mention that simple LE is also acceptable, Kenya only name LE ^20^, and Haiti do not mention any occlusion technique ^27^. The use of cautery is limited to four countries: Kenya, Rwanda, Haiti, and Colombia. Haiti and Kenya benefit from the support of No-Scalpel Vasectomy International (NSVI), a non-governmental organisation promoting and providing free NSV services in low-resource countries. In these two countries most vasectomies are done through NSVI. Thermal cautery, using a low-cost portable thermal cautery unit, combined with FI ^28^ is the vas occlusion technique recommended by NSVI (personal communication with Dr. Doug Stein, President of NSVI). In Rwanda, mucosal cautery of the vas combined with FI ^28^ has been successfully introduced in 2010 ^29^ and is now recommended to be used for occluding the vas ^21^. Profamilia in Colombia has recently introduced thermal cautery combined with FI ^28^ as one of their recommended techniques, in addition to LE+FI ^26^. They aim to train all urologists from their family planning clinic network over year 2019 (personal communication with Dr. Diana Torres, chief urologist at Profamilia). Colombia is then the only one of the four large vasectomy programs to recommend using cautery ( Table 2).

## Discussion

Creating and sustaining successful vasectomy programs in low-resource countries is challenging. Demand for vasectomy, access to services, and enabling environment must all be mutually reinforced ^13^. Skillful vasectomy providers performing best practice surgical techniques is an essential component contributing to the success of vasectomy programs in countries where acceptance of vasectomy is low, follow-up of patients for complications is difficult, and access to post-vasectomy semen analysis to confirm success (or failure) of the procedure is not available.

On one hand, as recommended in the evidence-based vasectomy guidelines, NSV is uniformly adopted in the selected low-resource countries for isolating the vas deferens, minimizing the risk of bleeding and infection. On the other hand, cautery, which is recommended for occluding the vas in the guidelines, is seldom encountered in the targeted countries. In these countries, the most common standard for occluding the vas is LE+FI.

Although no vasectomy occlusion technique has been shown to be superior in terms of contraceptive effectiveness in comparative trials ^9^, research evidence support the adoption of cautery over LE+FI for occluding the vas in low-resource settings ^4, 5, 30^. Occlusion failure risks of 2.1% ^31^ 2.5% ^32^, 2.6% ^33^, 5.9% ^2^ and 7.6% ^34^ have been reported for the LE+FI technique; these are much higher than the higher acceptable risk of occlusion failure of vasectomy, which is 1% ^10^. In addition, even if FI is recommended to be combined with LE to decrease failure rate, it may not be commonly performed. In 2004, it was estimated that more than 95%, 97%, and 99% of vasectomies were done with simple LE without FI in India, Nepal, and Bangladesh despite country standards ^35^. If no FI is added to LE, the occlusive failure risk is even higher and contraceptive failure may parallel occlusion failure. In a cohort of 1263 men from rural Nepal who had a vasectomy mostly performed by simple LE, 2.3% still had 500,000 sperm/ml or more in their semen 1 to 3 years after the procedure and the pregnancy rate reported was 4.2% after 3 years ^17^. Finally, modelling the cost per couple-years of protection of LE, LE+FI, cautery, and cautery + FI in India, Kenya, and Mexico showed that cautery-based techniques are the most cost-effective methods ^36^.

This study has two main limitations. First, the size of this convenience sample of eight countries is small. They were purposely chosen however to illustrate the situation in large and small vasectomy programs located on three continents. Second, some of the documents reviewed may be outdated. It is very only recently that Profamilia in Colombia updated their standards to include cautery combined with FI as the preferred occlusion technique of the vas ^26^. To the author’s knowledge, Haiti, Nepal, and Mexico are currently updating their male sterilization norms and standards. A future assessment of the norms and standards of the targeted countries and other low-resource countries with active vasectomy program may yield different results.

In conclusion, in low-resource countries, NSV is largely adopted for vas isolation in accordance with evidence-based guidelines but recommended techniques for vas occlusion are not. Providing the most effective vasectomy surgical techniques increase users’ confidence and satisfaction regarding male sterilization ^13^ and may lead to higher acceptability and increase uptake.

## Data availability

All data underlying the results are available as part of the article and no additional source data are required.
