# Supplementary material for: Are evidence-based vasectomy surgical techniques performed in low-resource countries?
Source: Gates Open Res. 2019 Jul 22;3:1462. Originally published 2019 May 10. [Version 2] doi: 10.12688/gatesopenres.12986.2 (PMC6584738; doi:10.12688/gatesopenres.12986.2)
Supplement: Peer review history

# Peer review: Reviewer response for version 1

**DOI:** 10.21956/gatesopenres.14093.r27345

**Published:** 2019-07-10

**Authors:** Joel L. Marmar

**Affiliations:** 1 Honorary Staff, Cooper University Hospital, Camden, NJ, USA

In this report, M. Labrecque, M.D., provided specific information and data on vasectomies based on national and organizational vasectomy guidelines to determine the utilization and risks of vasectomies in high and low-resource countries. Overall, he evaluated the effectiveness, safety and outcome of these procedures. Thus, it seems reasonable that this information may stimulate development of improved vasectomy techniques, and potentially lead to more male participation in family planning.

 At first, the article reviewed the vasectomy guidelines that were published in high resource countries. They included the guidelines of the American Urologic Association (2012), European Urologic Association (2012), Faculty, Reproductive Healthcare of United Kingdom (2014), and the Urologic Association Guideline: Vasectomy (2016). In addition, Dr. Labrecque personally obtained and reviewed other vasectomy guidelines from 8 low resource countries, including: Kenya, Rwanda, India, Nepal, Mexico, Honduras, Colombia and Haiti. Despite the fact that vasectomies had significant potential for male family planning and population control in these countries, the question was whether vasectomies were utilized appropriately in these low resource countries, and whether vasectomies may reduce the need for the more risky and expensive female tubal ligations. 

 In the 4 advanced countries, the practice guidelines were evidence-based for the most part. They indicated that the access to the vas should be minimally invasive which means: 1) a skin opening of < 10mm, 2) minimal dissection of the vas and peri vasal tissues, and 3) no skin sutures. With this approach, there was high quality evidence for a lower risk of surgical complications, bleeding and hematomas. In addition, these guidelines suggested that occlusion of the vas was best accomplished with cautery of the mucosa within the vas lumen and the use of fascial interposition to cover the ends of the cut vas. With this combination, the results yielded the lowest failure rate of <1%.

 In contrast, it was interesting to note that only 6 of 8 of these low resource countries used a thermal cautery on a routine basis, and the use of fascial interposition was inconsistent. Most of these countries used excision and ligations of the cut ends of the vasa without fascial interposition, but this combination had a failure rate of 8-15%. When the cost/couple years of protection was computed, it indicated that cautery-based vasectomy techniques were the most cost-effective methods for family planning (Seamans and Harner-Jay, 2007 ^1^). Therefore, it seems that all vasectomies should probably include mucosa cauterization vas plus fascial interposition. In addition, more data should probably be accumulated to compare results and complications of vasectomies versus female tubal ligations, because these data may lead to more male participation in family planning with fewer failures and complications.

 In summary, Dr. Labrecque should be congratulated for his personal work effort. His current data on vasectomies in high and low resource countries should simulate further research for improved vasectomy techniques. Overall, if less expensive techniques are developed for mucosal cautery, and simpler methods are developed for fascial interposition, these new advances may produce even less invasive/more effective vasectomies which may reduce the need/risks of female tubal ligation. Bravo Dr. Labrecque.

I confirm that I have read this submission and believe that I have an appropriate level of expertise to confirm that it is of an acceptable scientific standard.

## References

[1] : Modelling cost-effectiveness of different vasectomy methods in India, Kenya, and Mexico. *Cost Eff Resour Alloc*.2007;5: 10.1186/1478-7547-5-8 8 10.1186/1478-7547-5-8 17629921PMC1947949

# Response

**Published:** 2019-07-17

**Authors:** michel labrecque

**Affiliations:** Université Laval, Canada

I wish to thank Dr. Marmar for his review.

# Peer review: Reviewer response for version 1

**DOI:** 10.21956/gatesopenres.14093.r27324

**Published:** 2019-06-19

**Authors:** Philip S. Li, Omar Al Hussein Alawamlh

**Affiliations:** 1 Center for Reproductive Medicine and Microsurgery, Department of Urology, Weill Cornell Medicine, New York City, NY, USA; 2 Department of Urology, Weill Cornell Medicine, New York City, NY, USA

**General:**

 In this article entitled “Are evidence-based vasectomy surgical techniques performed in low-resource countries?”, the author aimed to determine if evidence-based vasectomy surgical techniques are integrated in national vasectomy programs of selected 8 low-resource countries in Asia, South America and Africa after establishing which vasectomy techniques are recommended in evidence-based practice guidelines.

 This evidence-based article is well written, it clearly delineates the guidelines and data available on the techniques used for the vasectomy procedure of solation/explosion and occlusion of the vas deferens. Data indicated the preferred vasectomy technique in almost all of the select countries is the no-scalpel vasectomy (NSV), which happens to be the best surgical technique to isolate and expose the vas deferens with mucosal cautery (MC) of the vas, preferably combined with a small segment of the vas deferens along with fascial interposition (FI), and is the recommended and best surgical practice.

 The information presented is valuable in informing vasectomy services in low-resource countries, and that could improve outcomes and increase demand and uptake of vasectomy in those countries.

**Editorial comments: **

1. Page 3, 2 ^nd^ paragraph under introduction, 3 ^rd^ line: “1) what vasectomy surgical techniques …” should be changed to “1) which vasectomy surgical techniques …”.
2. Page 3, 1 ^st ^paragraph under results, 7 ^th^ line: “(known as the no-scalpel vasectomy (NSV)) should be perform to ...” should be changed to “(known as the no-scalpel vasectomy (NSV)) should be performed to …”.
3. Page 5, 3 ^rd^ paragraph under discussion, 2 ^nd^ line: “has been shown to be superior in term of contraceptive effectiveness …” should be changed to “has been shown to be superior in terms of contraceptive effectiveness …”.
4. Page 5, 4 ^th^ paragraph under discussion, 1 ^st^ line: “the sample of this convenience sample of eight countries is small.” should be changed to “the size of this convenience sample of eight countries is small.”

We confirm that we have read this submission and believe that we have an appropriate level of expertise to confirm that it is of an acceptable scientific standard.

# Response

**Published:** 2019-07-17

**Authors:** michel labrecque

**Affiliations:** Université Laval, Canada

I wish to thank Dr. Li and Dr. Al Hussein Alawamlh for their review. Grammatical errors noted were corrected in version 2.

# Peer review: Reviewer response for version 1

**DOI:** 10.21956/gatesopenres.14093.r27174

**Published:** 2019-06-06

**Authors:** David Sokal

**Affiliations:** 1 Male Contraception Initiative, Durham, NC, USA; 2 Adjunct Associate Professor, Department of Maternal and Child Health, UNC Gillings School of Public Health, Chapel Hill, NC, USA; 3 Member, American Andrology Society, Schaumburg, IL, USA

**General:**

- This is a very useful paper, and has implications for national vasectomy authorities, and for the World Health Organization, and for other organizations who wish to facilitate the use of best practices for vasectomy procedures. Support and training activities for vasectomy services deserve more attention.  

**Editorial comments: For clarity, please improve formatting and content of tables, specifically:**

 Table 1: 

- Put **“** *Vas isolation* **”** and **“** *Vas occlusion* **”** in bold, and/or better separate these categories in some other way(s). 
- Also, what and where is FSRH? Google tells me that it is a UK standards body. That should be noted. 

Table 2:

- Italics is not sufficient to clearly identify the large and small programs, and ordering the countries by region seems less useful than ordering them by large and small, or put the large programs in bold?

*Chair of the Board at the Male Contraceptive Initiative (MCI), Durham, NC, USA. This is an unpaid volunteer position. This review reflects my personal views, and not those of MCI.*

I confirm that I have read this submission and believe that I have an appropriate level of expertise to confirm that it is of an acceptable scientific standard.

# Response

**Published:** 2019-07-17

**Authors:** michel labrecque

**Affiliations:** Université Laval, Canada

I wish to thank Dr. Sokal for his review. Although I agree that a bold format for vasectomy techniques (table 1) and countries (table 2) would enhance clarity, the current format was suggested by the editor. I clarified who FRSH are in the text: the Faculty of Sexual & Reproductive Healthcare (FSRH), the standard-setting organisation for family planning and sexual health in United Kingdom (2104).
